# Nonlinearity association between hyperuricemia and all-cause mortality in patients with chronic kidney disease

**DOI:** 10.1038/s41598-023-51010-6

**Published:** 2024-01-05

**Authors:** Ya-Fei Liu, Liang Han, Yin-Hong Geng, Huan-Huan Wang, Jia-Hui Yan, Sheng-Hao Tu

**Affiliations:** 1 Department of Nephrology, The First Affiliated Hospital of Zhengzhou University, 1 Jianshe East Road, Zhengzhou, 450052 Henan China; 2 Institute of Integrated Traditional Chinese and Western Medicine, Tongji Hospital, Tongji Medical College, Huazhong University of Science and Technology, 1095 Jiefang Avenue, Wuhan, 430030 Hubei China

**Keywords:** Endocrinology, Nephrology

## Abstract

Controversy surrounds the role of serum uric acid and whether treatment intervention is favorable in retarding the progression of chronic kidney disease (CKD). The association of serum uric acid levels and CKD patient mortality risk needs to be further determined by large sample cohort studies. The National Health and Nutrition Examination Survey participants with CKD from 1998 to 2017 were enrolled in the study. Multivariable Cox regression models were used to reveal the association of serum uric acid concentrations and CKD mortality risks. A total of 9891 CKD patients were enrolled in the study, and 3698 individuals died during the follow-up. Increasing serum uric acid levels are independently relevant to higher mortality risks of CKD patients (HR per SD increase). A restricted cubic spline curve showed a nonlinear association between serum uric acid and CKD mortality risks (*p* for nonlinearity = 0.046). CKD patients with higher levels of serum uric acid (≥ 5.900 mg/dL) show a significant increase in mortality risks (HR = 1.102, 95% CI 1.043–1.165). Sensitivity analysis demonstrated that the results were stable and robust. High serum uric acid levels (≥ 5.900 mg/dL) may be associated with increased mortality risks in CKD patients.

## Introduction

Uric acid is the final product of purine metabolism in humans due to a lack of uricase, and 70% is excreted through the kidneys. Disorders of purine metabolism, increased uric acid production, and decreased excretion can all lead to an imbalance of uric acid, resulting in a series of complications, such as gout^1^, kidney stones, metabolic syndrome^2^, hypertension^3^, cardiovascular diseases^4^, and progressive chronic kidney disease (CKD)^5^. With the continuous improvement of living standards and changes in people's eating habits, the prevalence of hyperuricemia is rising, which has attracted individuals’ attention^6^.

CKD is an irreversible, progressive condition characterized by chronic loss of kidney function and structural damage to the kidneys. CKD has become a major public health problem worldwide^7^. In the United States and China, the prevalence of CKD is as high as 10.2% and 10.8%, respectively^8,9^. In patients with kidney diseases, it is known that uric acid levels also rise as kidney damage progresses. It is assumed that elevated uric acid levels in kidney patients are not only a consequence of kidney damage but may also worsen kidney damage, leading to poor prognosis and even death.

However, it should be noted that the effect of uric acid on CKD progression and CKD patient mortality has long been controversial^10–15^. It has been proposed that uric acid is an independent predictor of early renal failure and has a J-type relationship with all-cause mortality in CKD^16,17^. In nondiabetic CKD, the association between uric acid levels and the risk of mortality appears to be influenced by the estimated glomerular filtration rate (eGFR)^18^. Furthermore, an analysis using longitudinal data suggests that an increasing trajectory of uric acid is associated with accelerated renal failure and all-cause mortality in CKD patients^19^. However, another similar analysis using longitudinal data indicated a strong nonlinear association between longitudinal uric acid levels and the risk of renal failure and mortality in CKD patients^20^. A small number of randomized controlled trials (RCTs) have demonstrated that lowering serum uric acid with xanthine oxidase inhibitors facilitates slowing the progression of CKD^21–23^. Several single-center trials have shown that allopurinol or febuxostat decelerate the progression of CKD over a follow-up of 6 to 12 months^21,23,24^. In contrast, the results of Kim et al. showed that higher uric acid was associated with lower all-cause mortality^25^. In addition, an interesting study indicated that asymptomatic hyperuricemia was not an independent risk factor for CKD progression^26^. There was a significant but much weaker association between greater uric acid levels and incident chronic kidney disease^27^. A meta-analysis indicated that urate-lowering therapy (ULT) may make little or no difference in the incidence of kidney failure or death in participants with or without CKD^28^.

Previous studies are uncertain of the effectiveness of ULT on CKD. Coupled with the growing recognition of the importance of the link between hyperuricemia and CKD, large sample studies are needed to investigate the impact of uric acid on the progression of CKD and whether ULT can delay the progression of CKD and improve poor prognosis. Answering these questions may have a significant impact on the prevention and control of CKD in the future.

## Methods

### Study design and enrolled population

**Figure 1:**
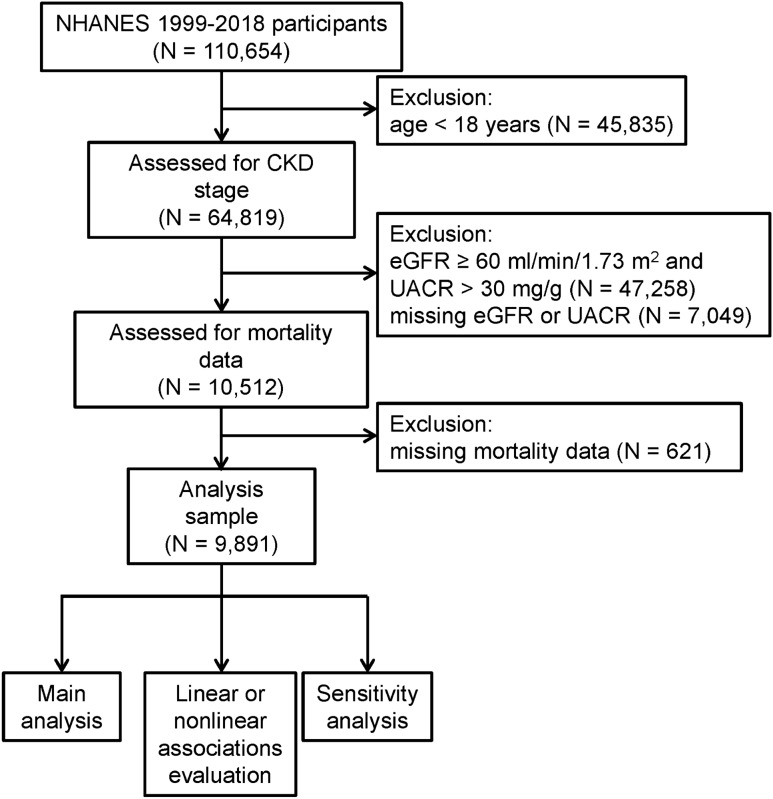
Flowchart of study participants. NHANES: National Health and Nutrition Examination Survey, CKD: chronic kidney disease, eGFR: estimated glomerular filtration rate, UACR: urinary albumin‒creatinine ratio.

The National Health and Nutrition Examination Survey (NHANES) is a recurring cross-sectional survey planned and performed by the National Center for Health Statistics (NCHS) of the Centers for Disease Control and Prevention (CDC) of the United States (www.cdc.gov/nchs/nhanes/index.htm.). The survey is designed to monitor the health and nutritional status of the whole nation via a complex weighted survey design and large-scale questionnaire, physical examination and laboratory investigations. The NHANES has been conducted in 2-year survey cycles since 1999. The protocol of NHANES was approved by the NCHS Research Ethics Review Board. Written informed consent was obtained from all participants (www.cdc.gov/nchs/nhanes/irba98.htm.). All data used for analysis in the present study can be accessed and downloaded from the website (https://wwwn.cdc.gov/nchs/nhanes/search/datapage.aspx). The ethical review of this study was exempted by the Institutional Review Board of the author's institution (the Ethics Committee of the First Affiliated Hospital of Zhengzhou University). All methods in our research were performed in accordance with the Declaration of Helsinki. Ten cycles of NHANES were downloaded from 1999 to 2018. Individuals under the age of 18 were initially excluded. Subsequently, the eGFR of individuals aged 18 and above was calculated via the CKD Epidemiology Collaboration (CKD-EPI) creatinine equation. Individuals with eGFR < 60 ml/min/1.73 m^2^ or urinary albumin‒creatinine ratio (UACR)  > 30 mg/g were considered CKD patients^29^. Participants were evaluated to ensure that they had chronic kidney injury according to the levels of eGFR and UACR. CKD stage was also further divided via eGFR and UACR levels (Supplementary Table S1)^29^. Due to the lack of available renal tissue biopsy results, we only used eGFR and UACR for staging CKD patients. The details of the detection methods for creatinine and UACR will be described in the Methods section, specifically in the assessment of covariates. Only individuals with stages 1–5 CKD were retained. Finally, individuals without serum uric acid and all-cause mortality data were screened out. An overview of the entire study process can be found in Fig. 1.

### Serum uric acid detection

After collection, the blood samples obtained from the participants were appropriately processed and either refrigerated or frozen. The blood samples were then utilized and subjected to the necessary testing in the designated laboratory as specified by NHANES. A colorimetric method was applied to detect serum uric acid. According to the NHANES suggestion, the serum uric acid level in NHANES 2017–2018 was adjusted before analysis via the nonweighted Deming regression method. Blood uric acid levels were assessed using validated protocols and procedures. Specific testing protocols can be found at https://www.cdc.gov/nchs/nhanes.

### Mortality data

Death certificate records from the National Death Index (NDI) have been linked to individuals who participated in the NHANES. The mortality follow-up data were available until December 31, 2019. Data on total all-cause mortality were obtained from https://www.cdc.gov/nchs/data-linkage/mortality.htm.

### Covariates

In the NHANES, the age, sex, and race of individuals were documented during demographic surveys via questionnaires. Age was included as a categorical variable because individuals in NHANES older than 80 or 85 years were also recorded as 80 or 85 years, respectively. The race of enrolled individuals was classified as Mexican American, Other Hispanic, Non-Hispanic White, Non-Hispanic Black, and others. Individuals with multiple racial backgrounds have also been classified as others. All individuals’ body mass index was calculated and divided into underweight (BMI < 18.5), normal (18.5 ≤ BMI < 24.5), overweight (24.5 ≤ BMI < 30), and obese (BMI ≥ 30). The participants' educational level (College or higher, high school, and less than high school) and marital status (Married/cohabiting, Never married, Widowed/divorced/separated) were obtained through a questionnaire survey.

Surveys via questionnaires were also applied to evaluate whether the individuals had hypertension and diabetes via two questions: “Has a doctor or other health professional told you had high blood pressure?” and “Has a doctor or other health professional told you had diabetes or sugar diabetes?” Participants' history of cardiovascular diseases (CVD) was determined by the following questions: "Have you ever been told you had congestive heart failure? ", "Have you ever been told you had coronary heart disease? ", "Have you ever been told you had angina/angina pectoris? ", and "Have you ever been told you had a heart attack?". The participants' dietary habits were assessed through a questionnaire survey to gather information on their intake of energy, carbohydrates, proteins, total fat, and dietary fiber over the past 24 h. Information on participants' prescription medication use in the month prior to the interview date was collected through a questionnaire. The usage of uric acid-lowering medications was recorded, along with other medications that may affect the risk of mortality in CKD patients, such as angiotensin-converting-enzyme inhibitors (ACEIs), angiotensin receptor blockers (ARBs), sodium-glucose transport protein 2 (SGLT2) inhibitors, angiotensin receptor neprilysin inhibitors (ARNIs), and mineralocorticoid receptor antagonists (MRAs).

Considering the different levels of uric acid in patients with different severities of CKD, CKD stage, albumin to globulin ratio (AGR), blood albumin level and glycohemoglobin (HbA1c) were also included as covariates. As mentioned earlier, the CKD stage is determined based on eGFR and UACR levels (Supplementary Table S1). The participants' eGFR was estimated using the CKD-EPI creatinine equation. Blood creatinine and albumin levels were obtained and stored using the same methods as serum uric acid. Creatinine in blood was measured using either the Jaffe rate method or enzymatic method, while albumin levels in blood samples were measured using the bichromatic digital endpoint method or the dye bromocresol purple (BCP) method. HbA1c levels were measured using high-performance liquid chromatography (HPLC). Total protein levels in blood were measured using the timed rate biuret method or biuret reaction, and the globulin level was obtained by subtracting the albumin level from the total protein level. Random urine specimens were collected at the survey mobile examination center, frozen, and stored at −70 °C upon arrival. Urine samples were analyzed in the designated laboratory of NHANES. Urinary albumin levels were measured using the solid-phase fluorescent immunoassay, and urinary creatinine levels were measured using either the Jaffe rate reaction or enzymatic method. Similar to the process for serum uric acid testing, validated protocols and procedures were utilized for the assessment of blood creatinine, blood albumin, AGR, urinary albumin, and urinary creatinine. According to the NHANES analytic notes, any impact of instrument or method changes on measurement data has been corrected using specific regression equations. Finally, NHANES cycles were also included as a covariate to exclude the potential influence produced by different survey cycles.

### Statistical analysis

The study visit weight, primary sampling unit (PSU) and stratification design of each sample were downloaded at the same time and were considered during constructing the Cox regression model by using the R package “survey” and its dependent packages. The χ^2^ test and Mann‒Whitney U test were utilized for categorical variables and continuous variables, respectively. Three Cox regression models were constructed to reveal the relationship of serum uric acid levels and CKD mortality risks. A crude model was first fitted without any covariates. Model 1 was adjusted for age and sex. In Model 2, race, education level, BMI, hypertension, diabetes history, smoking history, drinking history, eGFR, UACR and albumin, NHANES cycles, and covariates in Model 1 were included for additional adjustments. Serum uric acid levels were further categorized in Model 1 and included for additional adjustments. Serum uric acid levels were further categorized into quintiles (quintile 1 through quintile 5). In each quintile, CKD mortality risk was calculated. In addition, a test for trend was performed by employing the median value of each category of serum uric acid as a continuous variable. Restricted cubic splines with 3 knots illustrated by the “rms” package were employed to evaluate the potential linear or nonlinear associations. If there were nonlinear relationships between serum uric acid and CKD mortality, the recursive algorithm was used to calculate the inflection points.

Sensitivity analysis was first performed by stratified analysis. Additionally, patients who passed away within two years and patients without available covariate data were excluded from the model for additional sensitivity analyses. Additionally, CVD status, HbA1c levels, and the use of relevant medications were included as additional covariates in the model to assess the potential impact of CVD, HbA1c levels, and medication use on the risk of mortality in CKD patients. Since the 1999–2000 survey cycle did not include results for HbA1c, only data from the remaining nine cycles were included in the sensitivity analysis.

## Results

### Demographics and baseline characteristics

In the present study, a total of 9891 CKD patients (56.1% over 60 years old and 57.8% female) were enrolled. Table 1 illustrates the demographics and baseline characteristics of 9891 individuals according to whether they died. Among all CKD patients, the majority had stage 1 to stage 3 CKD, similar to previous research findings^29^. Compared with living participants, dead individuals were skewed toward older age and had worse CKD stages, lower eGFR and higher serum uric acid concentrations. Moreover, dead individuals with CKD exhibited a higher baseline proportion with hypertension or diabetes. However, the discrimination of BMI was not obvious.

**Table 1:** Baseline characteristics of 9891 chronic kidney diseases (CKD) patients in National Health and Nutrition Examination Survey (NHANES).

Characteristics	Overall	Alive	Dead	*P* value
n	9891	6193	3698	
Age				< 0.001
18–60 years	3498 (43.9)	3119 (57.6)	379 (15.6)	
≥ 60 years	6393 (56.1)	3074 (42.4)	3319 (84.4)	
Sex				< 0.001
Female	5270 (57.6)	3520 (59.3)	1750 (54.0)	
Male	4621 (42.4)	2673 (40.7)	1948 (46.0)	
Race				< 0.001
Non-Hispanic White	4418 (66.7)	2221 (61.5)	2197 (77.4)	
Non-Hispanic Black	2258 (14.4)	1735 (15.4)	823 (12.3)	
Mexican American	1485 (7.3)	1073 (9.1)	412 (3.5)	
Other Hispanic	696 (5.2)	553 (6.3)	143 (2.9)	
Others	734 (6.5)	611 (7.7)	123 (3.9)	
Education				
College or higher	3990 (48.2)	2758 (52.7)	1232 (39.0)	
High school	2360 (25.9)	1486 (25.7)	874 (26.5)	
Less than high school	3516 (25.7)	1940 (21.6)	1576 (34.1)	
Unknown	25 (0.2)	9 (0.1)	16 (0.3)	
Marital status				
Married/cohabiting	5050 (54.6)	3332 (58.0)	1718 (47.8)	
Never married	1132 (11.4)	927 (14.1)	205 (5.8)	
Widowed/divorced/separated	3477 (31.9)	1763 (25.7)	1714 (44.7)	
Unknown	232 (2.1)	171 (2.3)	61 (1.7)	
Smoking history				
Current	1667 (17.5)	1080 (18.0)	587 (16.5)	
Former	3174 (31.8)	1649 (27.5)	1525 (40.7)	
Never	4790 (49.0)	3213 (52.2)	1577 (42.5)	
Not available	260 (1.6)	251 (2.2)	9 (0.3)	
Drinking history				
Heavy drinker	1712 (17.8)	1053 (17.4)	659 (18.6)	
Low-to-moderate drinker	4145 (47.2)	2865 (52.2)	1280 (36.9)	
Non-drinker	1609 (14.5)	953 (12.9)	656 (17.9)	
Not available	2425 (20.6)	1322 (17.6)	1103 (26.7)	
Hypertension				< 0.001
Yes	5956 (56.7)	3360 (50.5)	2596 (69.5)	
No	3898 (42.9)	2815 (49.3)	1083 (29.7)	
Unknown	25 (0.3)	10 (0.1)	15 (0.6)	
Missing	12 (0.1)	8 (0.1)	4 (0.1)	
Diabetes				< 0.001
Yes	2875 (25.1)	1622 (21.6)	1253 (32.1)	
No	6740 (72.1)	4407 (75.6)	2333 (65.0)	
Borderline	265 (2.7)	158 (2.7)	107 (2.8)	
Unknown	11 (0.1)	6 (0.1)	5 (0.1)	
Cardiovascular diseases				< 0.001
Yes	(24.2)	(15.8)	(41.5)	
No	(73.1)	(80.9)	(57.0)	
Unknown	(2.7)	(3.3)	(1.5)	
BMI, kg/m^2^				< 0.001
Underweight	221 (2.4)	138 (2.4)	83 (2.4)	
Normal range	2090 (22.0)	1232 (21.5)	858 (23.2)	
Overweight	3260 (30.8)	1949 (30.3)	1257 (31.9)	
Obese	4041 (41.9)	2784 (44.6)	1257 (36.5)	
Not available	333 (2.8)	90 (1.3)	243 (6.1)	
Serum uric acid, mg/dL	5.80 [4.70, 6.95]	5.70 [4.60, 6.80]	6.20 [5.10, 7.40]	< 0.001
CKD stage				< 0.001
Stage 1	3219 (36.7)	2748 (46.6)	471 (16.4)	
Stage 2	2240 (21.6)	1302 (20.4)	938 (24.0)	
Stage 3a	2771 (27.6)	1455 (24.2)	1316 (34.4)	
Stage 3b	1138 (10.1)	493 (6.4)	645 (17.6)	
Stage 4	342 (2.7)	121 (1.5)	221 (5.2)	
Stage 5	181 (1.3)	74 (0.8)	107 (2.4)	
eGFR, mL/min/1.73m^2^	72.88 [53.11, 100.95]	86.46 [57.06, 108.01]	56.75 [44.86, 78.92]	< 0.001
UACR, mg/g	45.37 [24.89, 101.82]	45.39 [30.26, 92.93]	45.17 [17.40, 121.42]	0.245
Creatinine, mg/dL	1.00 [0.77, 1.27]	0.92 [0.73, 1.19]	1.13 [0.90, 1.40]	< 0.001
Urinary albumin, μg/mL	42.50 [15.10, 114.60]	46.50 [16.20, 111.14]	36.98 [13.10, 119.00]	
Albumin				< 0.001
Normal (≥ 3.5 g/dL)	9536 (97.1)	6030 (98.0)	3506 (95.3)	
Low (< 3.5 g/dL)	355 (2.9)	163 (2.0)	192 (4.7)	
AGR				
Normal (≥ 1)	9204 (95.0)	5858 (96.2)	3506 (92.3)	
Low (< 1)	677 (4.9)	325 (3.6)	352 (7.7)	
Not available	10 (0.1)	10 (0.2)	0 (0.0)	
HbA1c, %	5.70 [5.30, 6.30]	5.60 [5.30, 6.20]	5.80 [5.40, 6.50]	< 0.001
Intakes during past 24 h				
Energy, kcal	1737.00 [1285.00, 2318.00]	1830.28 [1357.00, 2439.91]	1576.00 [1192.09, 2105.14]	
Carbohydrate, g	210.67 [154.15, 284.15]	219.76 [161.61, 299.31]	195.63 [143.58, 258.05]	
Protein, g	65.13 [47.15, 90.58]	69.10 [49.19, 93.73]	59.82 [44.60, 82.12]	
Total fat, g	63.68 [42.40, 92.38]	67.86 [44.57, 97.17]	57.64 [39.18, 82.85]	
Dietary fiber, g	13.10 [8.50, 19.36]	13.40 [8.60, 20.10]	12.59 [8.30, 18.00]	
NHANES cycle				< 0.001
1999–2000	803 (7.8)	286 (5.1)	517 (13.4)	
2001–2002	887 (8.8)	365 (5.9)	522 (14.8)	
2003–2004	876 (8.6)	342 (6.0)	534 (13.8)	
2005–2006	848 (8.9)	435 (7.4)	413 (12.0)	
2007–2008	1006 (8.8)	566 (8.1)	440 (10.1)	
2009–2010	926 (8.0)	597 (8.2)	329 (7.7)	
2011–2012	1774 (18.7)	1234 (19.8)	540 (16.4)	
2013–2014	938 (10.5)	730 (12.4)	208 (6.5)	
2015–2016	881 (9.7)	752 (12.7)	129 (3.7)	
2017–2018	952 (10.2)	886 (14.4)	66 (1.6)	
Drug usage				
Uric acid lowering agent	(5.5)	(4.7)	(7.0)	0.004
ACEI	(22.5)	(19.4)	(28.9)	< 0.001
ARB	(11.8)	(11.2)	(13.0)	0.081
MRA	(1.8)	(1.3)	(3.0)	< 0.001
SGLT2	(1.6)	(1.7)	(1.5)	0.724
ARNI	(0.0)	(0.1)	(0.0)	0.136

Data were expressed as the n (%) or median [IQR]. Percentages were adjusted for NHANES complex survey design. χ^2^ test and Mann–Whitney U test were utilized for categorical variables and continuous variables respectively.

*CKD* chronic kidney disease, *eGFR* estimated glomerular filtration rate, *UACR* urinary albumin-creatinine ratio, *AGR* albumin/Globulin Ratio, *NHANES* National Health and Nutrition Examination Survey.

### Relationship between serum uric acid and CKD mortality

There was a total of 3698 individuals who died for 9891 CKD enrolled patients during approximately 20 years of follow-up. Overall, higher serum uric acid levels correspond to a higher risk of mortality in CKD patients after controlling for confounding variables (hazard ratios (HR) = 1.073, 95% confidence intervals (95% CI): 1.022–1.127) (Table 2). To investigate the association of different serum uric acid levels and mortality risk in CKD patients in more detail, 9891 individuals were labeled according to the quintiles of serum uric acid concentrations. Further Cox regression analysis indicated that in the crude model, higher serum uric acid levels were associated with higher mortality risk in quintile 2–5 CKD patients than in quintile 1 CKD patients, which was particularly evident in quantile 5 CKD patients. However, after adjusting for confounding variables, the HR and 95% CI of quintiles 1–4 were 1 (reference), 1.038 (0.903–1.193), 1.013 (0.822–1.116) and 1.013 (0.876–1.184), respectively. Meanwhile, the mortality risks of quintile 5 CKD patients still robustly increased (HR = 1.203, 95% CI 1.016–1.425). The trend test suggested a significant trend between CKD mortality and serum uric acid concentrations (*P* for trend = 0.014) after adjusting for the related confounding variables.

**Table 2:** Associations of serum uric acid level with mortality among 9891 CKD patients.

Mortality	Serum uric acid levels					*P* for trend	Per serum uric acid SD increment
	≤ 4.500 mg/dL (Q1)	4.521 mg/dL—5.360 mg/dL (Q2)	5.400 mg/dL—6.293 mg/dL (Q3)	6.300 mg/dL—7.225 mg/dL (Q4)	≥ 7.300 mg/dL (Q5)		
Crude model	1 (reference)	1.446 (1.248–1.675)	1.430 (1.230–1.663)	1.702 (1.454–1.993)	2.392 (2.059–2.779)	< 0.001	1.280 (1.235–1.326)
Model 1	1 (reference)	1.139 (0.997–1.301)	1.008 (0.867–1.171)	1.167 (1.002–1.361)	1.501 (1.296–1.739)	< 0.001	1.151 (1.104–1.200)
Model 2	1 (reference)	1.038 (0.903–1.193)	0.958 (0.822–1.116)	1.013 (0.867–1.184)	1.203 (1.016–1.425)	0.014	1.073 (1.022–1.127)

Crude model: without adjustment.

Model 1: adjusted for age (categorial) and sex.

Model 2: adjusted for model 1 plus race, education, marital status, smoking history, drinking history, dietary intakes during the past 24 h (continuous), body mass index (categorial), hypertension, diabetes, albumin (categorial), albumin/globulin ratio (categorial), urinary albumin level (continuous), chronic kidney disease stages (categorial) as well as National Health and Nutrition Examination Survey cycle.

**Figure 2:**
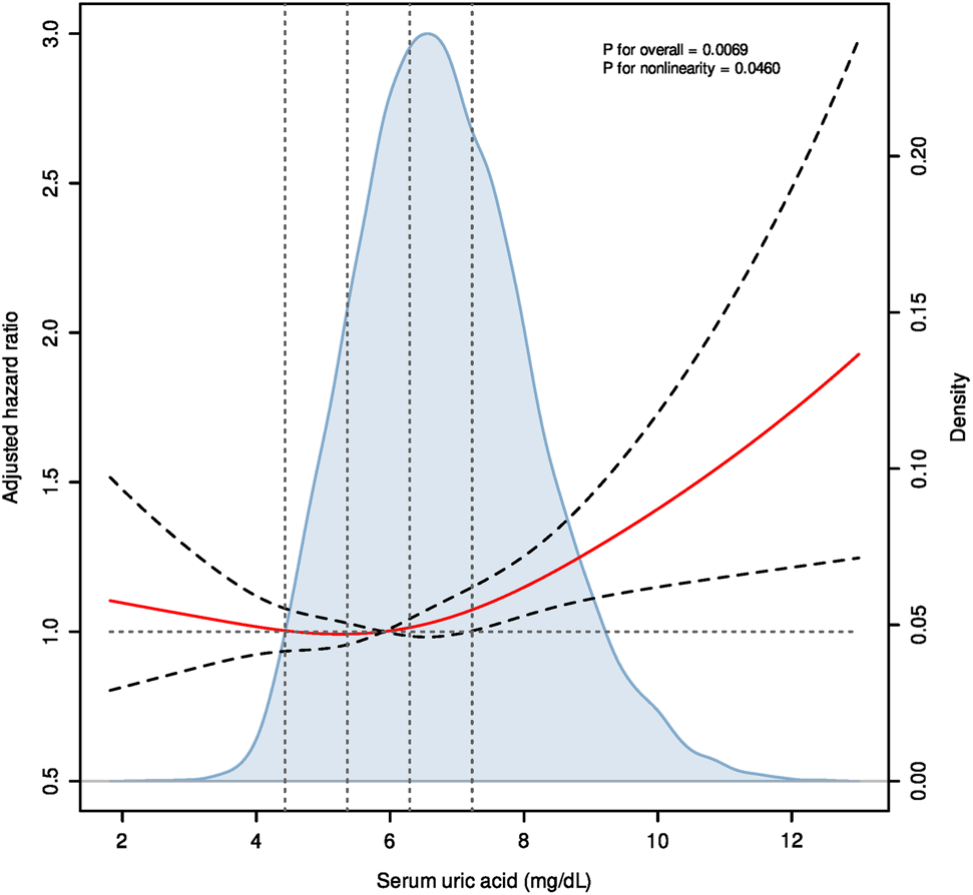
Dose‒response curves for serum uric acid levels and mortality. Hazard ratio (HR) (labeled as red curves) and 95% confidence intervals (labeled as black dashed curves) were adjusted for age, sex, race, body mass index (categorial), hypertension, diabetes, albumin (categorial), albumin/globulin ratio (categorial), chronic kidney disease stages (categorial) and National Health and Nutrition Examination Survey cycle. The horizontal dotted line represents the reference HR, and vertical dotted lines are illustrated according to the quintile of serum uric acid concentrations. The blue-shaded area indicates the probability density distribution of serum uric acid concentrations. To decrease the influence of outliers, individuals with the ten largest and ten smallest serum uric acid concentrations were removed before illustration.

We then attempted to evaluate the dose‒response relationship between serum uric acid concentrations and mortality risks of CKD patients through restricted cubic splines. Similar to the results of trend tests, growing serum uric acid levels were associated with increasing mortality risks among CKD patients overall (Fig. 2). The curve appears as a slow J shape without a hook instead of a linear shape, and the nonlinearity tests also suggest the nonlinear trend of the spline (*P* for nonlinearity = 0.046). According to the curves, the mortality risks of CKD patients in quintiles 4 and 5 start to increase dramatically with increasing serum uric acid concentrations, especially in quintile 5. The mortality risks of CKD patients in quintiles 1–3 of serum uric acid were close to the reference level. We then determined that the inflection point of serum uric acid was 5.9 mg/dL via a recursive algorithm.

In further analysis, we divided the patients into 2 subgroups according to the inflection points. Models were separately constructed for the two subgroups. The results demonstrated that CKD patients with higher serum acid levels had a significant increase in mortality risk (HR = 1.102, 95% CI 1.043–1.165) but not CKD patients with lower levels (HR = 0.958, 95% CI 0.884–1.037) (Table 3).

**Table 3:** Associations of lower and higher than inflection points of serum uric acid level with mortality among CKD patients.

Mortality	Serum uric acid < 5.9 mg/dL	Serum uric acid ≥ 5.9 mg/dL	Overall
Crude model	1.225 (1.135–1.323)	1.196 (1.149–1.246)	1.189 (1.160–1.219)
Model 1	0.951 (0.885–1.021)	1.177 (1.124–1.233)	1.104 (1.072–1.137)
Model 2	0.958 (0.884–1.037)	1.102 (1.043–1.165)	1.051 (1.016–1.088)

Crude model: without adjustment.

Model 1: adjusted for age (categorial) and sex.

Model 2: adjusted for model 1 plus race, education, marital status, smoking history, drinking history, dietary intakes during the past 24 h (continuous), body mass index (categorial), hypertension, diabetes, albumin (categorial), albumin/globulin ratio (categorial), urinary albumin level (continuous), chronic kidney diseases stages (categorial) as well as National Health and Nutrition Examination Survey cycle.

### Sensitivity analyses

Sensitivity analysis was performed to estimate the robustness of the models. First, through stratification analyses, we found that there was no significant interaction when stratified by age, sex, race, BMI, hypertension, diabetes or CKD stages, which indicated that the results from Cox regression models were quite robust (Supplementary Table S2). Subsequently, we eliminated individuals who died within the first two years and reconstructed the Cox regression models; new models suggested that the association of serum uric acid concentrations and mortality risks among CKD patients was not influenced by the elimination (Supplementary Table S3). Afterward, a small proportion of individuals had missing or no available records of history of hypertension, diabetes, AGR and BMI. We attempted to remove these individuals from the newly constructed models, and the results also revealed that they did not affect the conclusion (Supplementary Table S4). Finally, when CVD status, HbA1c levels, and the usage of uric acid-lowering medications and other relevant drugs potentially improving renal damage were additionally included in the model, it was found that the results did not show significant changes (Supplementary Tables S5 and S6). It is important to note that after incorporating medication usage into the model, the relative risk of mortality among CKD patients across different uric acid levels could not be calculated. Therefore, the analysis results for this aspect could not be fully presented.

## Discussion

Overall, the role of serum uric acid levels in CKD progression and prognosis remains controversial and is still being debated. It has been proven that uric acid can destroy vascular endothelial function by reducing endothelial nitric oxide synthase^30^. High levels of serum uric acid also induce the activation of the renin–angiotensin–aldosterone system (RAAS) and aggravate oxidative stress^31^. In addition to direct damage, hyperuricemia may lead to hypertension according to extensive evidence^32^, which may also influence the mortality risks of CKD patients. However, uric acid may also protect CKD patients against oxidative stress, which may contribute to the underlying beneficial effects on CKD patients.

Similar to the confusing biological mechanisms, many clinical studies would like to unravel whether serum uric acid influences progression and prognosis, but there are still no uniform conclusions about that. Recently, two randomized controlled trials investigated the impact of allopurinol treatment on the progression of CKD in patients with early to moderate diabetic nephropathy or stage 3 or 4 CKD^33,34^. The results of both clinical trials indicated that CKD patients may not benefit from allopurinol therapy. Therefore, these findings suggest that higher uric acid levels may not be a risk factor for CKD. Conflicting results influence the clinical decision, that is, whether urate-lowering therapy (ULT) should be applied to CKD patients. Therefore, more studies should be performed for further definitive conclusions.

In our population-based retrospective cohort study, a nonlinearity association was revealed by restricted cubic spline. Subsequently, the recursive algorithm suggests that the inflection point of the nonlinearity relationship is 5.9 mg/dL, which suggests that a high serum uric acid concentration (≥ 5.9 mg/dL) may strongly increase the mortality risk in CKD populations. Stratified analysis and sensitivity analysis demonstrated that our study findings have broad applicability to different stages or types of CKD patients, and the analysis results are robust. The results are not completely the same as the J-shaped mortality relationship for uric acid levels in 294 patients with CKD stage 5 starting renal replacement therapy^35^. Additionally, our results differ from those of a previous study that showed a U-shaped association between uric acid levels and cardiovascular disease mortality^36^. Hence, decreasing serum uric acid levels may be helpful to lower the mortality risks in CKD patients with high serum uric acid levels. However, as mentioned earlier, two RCTs do not support the notion that ULT improves disease progression in CKD patients. Moreover, in our study, even incorporating the use of uric acid-lowering medications as a covariate in the model did not affect the study conclusions. This suggests that the use of uric acid-lowering medications may not alter the positive association between uric acid levels and the risk of mortality in CKD patients. Whether low levels of serum uric acid concentrations affect the risk of CKD patient mortality is still controversial and needs further study.

According to the 2020 American College of Rheumatology guidelines for the management of gout, ULT is conditionally recommended for patients with CKD (stage ≥ 3), serum uric acid concentration > 9 mg/dL, or urolithiasis^37^. However, according to the 2019 guidelines for the diagnosis and management of hyperuricemia and gout in China, starting ULT is recommended for patients with serum uric acid concentration > 8 mg/dL and CKD (stage ≥ 2) or uric acid kidney stones and asymptomatic hyperuricemia with serum uric acid concentration > 9 mg/dL^38^. ULT initiation criteria and recommendation levels for CKD patients differed according to different guidelines. Meanwhile, serum uric acid levels are not always in parallel with CKD stages.

It is speculated that the reasons why uric acid increases the risk of mortality for CKD individuals include direct and indirect factors. Direct impairment of uric acid is made up of endothelial dysfunction, blocking the production of nitric oxide, inducing reactive oxygen species (ROS), Monocyte chemoattractant protein-1 (MCP-1) production, activation of RAAS, and insulin resistance^39^. Indirect impairment, such as hyperuricemia-related complications or comorbidities, including metabolic syndrome, atrial fibrillation^40^, heart failure^41^, cardiovascular disease, atherosclerosis^42^, and stroke^43^, could lead to the death of CKD patients.

This study has some highlights due to the innovation in four ways. First, despite CKD stages, the risk of mortality increases nearly with elevated serum uric acid. Second, trend tests and restricted cubic spline were used to explore the changing trends and dose‒response relationship between serum uric acid levels and risks of mortality in CKD patients, which were seldom applied in previously related studies. Moreover, compared to previous studies, the sample size of the present study is large enough to provide enough power for statistical analysis. Finally, our study includes stage 1 to 5 CKD patients, rather than just including potential CKD stages in some other studies, which suggests that the nonlinearity associations have somewhat broader applicability.

Our study had some shortcomings as well. First, the study only revealed the association between serum uric acid and mortality among CKD patients instead of a casual association. Second, data from the NHANES make the conclusions from our study not applicable to CKD patients in other regions. Third, the results from the Cox regression model may not be accurate due to the influence of known or unknown confounders that could not be fully controlled by multivariate Cox regression models. Fourth, our study population is not entirely consistent with other relevant studies, which may contribute to the partial inconsistency of our study results with other published related research. Last but not least, retrospective instead of prospective study design makes potential bias of data collection.

In conclusion, our study identified a nonlinear relationship between serum uric acid and mortality among CKD patients. CKD patients with high levels of serum uric acid (≥ 5.900 mg/dL) have higher mortality risks. However, the present study is an associative study, so it is difficult to distinguish between causation and association.

### Supplementary Information

Supplementary Information 1.

Supplementary Information 2.

Supplementary Information 3.

Supplementary Information 4.

Supplementary Information 5.

Supplementary Information 6.

## Data Availability

The raw data that supported the findings of this study are openly available in the public databases (https://wwwn.cdc.gov/nchs/nhanes/search/datapage.aspx).
