# Supplementary material for: Nonlinearity association between hyperuricemia and all-cause mortality in patients with chronic kidney disease
Source: Sci Rep. 2024 Jan 5;14:673. doi: 10.1038/s41598-023-51010-6 (PMC10770354; doi:10.1038/s41598-023-51010-6)
Supplement: Supplementary file 1 — Supplementary Information 1. [file 41598_2023_51010_MOESM1_ESM.docx]

Supplementary Table 1: Definition of chronic kidney disease (CKD) stage.

| **Stages** | **eGFR** | **UACR** |
| --- | --- | --- |
| Stage 1 | ≥ 90.0 | ≥30 |
| Stage 2 | 60.0-89.9 | ≥30 |
| Stage 3a | 45.0-59.9 | - |
| Stage 3b | 30.0-44.9 | - |
| Stage 4 | 15.0-29.9 | - |
| Stage 5 | <15.0 | - |
| Without CKD | Individuals who did not meet any CKD stages. | |

Individuals who meet the criterion of eGFR and UACR simultaneously were divided into the corresponding stages.

eGFR: estimated glomerular filtration rate, UACR: urinary albumin-creatinine ratio.
