# Supplementary material for: Nonlinearity association between hyperuricemia and all-cause mortality in patients with chronic kidney disease
Source: Sci Rep. 2024 Jan 5;14:673. doi: 10.1038/s41598-023-51010-6 (PMC10770354; doi:10.1038/s41598-023-51010-6)
Supplement: Supplementary file 2 — Supplementary Information 2. [file 41598_2023_51010_MOESM2_ESM.docx]

**Supplementary Table 2: Stratified analyses of the associations of serum uric acid levels with mortality among CKD patients.**

|  | **HR (95% CI)** | ***P* value for interaction** |
| --- | --- | --- |
| **Age, years** |  |  |
| 18-60 years | 1.080 (0.985-1.185) | 1.000 |
| ≥60 years | 1.047 (1.009-1.087) |  |
| **Sex** |  |  |
| Female | 1.056 (1.007-1.107) | 1.000 |
| Male | 1.033 (0.982-1.086) |  |
| **Race** |  |  |
| Non-Hispanic White | 1.049 (1.009-1.091) | 1.000 |
| Non-Hispanic Black | 1.113 (1.053-1.176) |  |
| Mexican American | 1.042 (0.941-1.155 |  |
| Other Hispanic | 1.141 (0.967-1.345) |  |
| Others | 0.854 (0.715-1.021) |  |
| **Education** |  |  |
| College or higher | NA | 1.000 |
| High school | 1.084 (1.003-1.170) |  |
| Less than high school | 1.048 (0.997-1.103) |  |
| Unknown | NA |  |
| **Marital status** |  |  |
| Married/cohabiting | 1.078 (1.028-1.131) | 1.000 |
| Never married | 1.051 (0.903-1.223) |  |
| Widowed/divorced/separated | 1.027 (0.977-1.079) |  |
| Unknown | 0.836 (0.626-1.115) |  |
| **BMI** |  |  |
| Underweight | 1.274 (0.836-1.942) | 1.000 |
| Normal range | 1.018 (0.938-1.104) |  |
| Overweight | 1.057 (1.000-1.118) |  |
| Obesity | 1.047 (0.993-1.103) |  |
| Not available | 0.980 (0.869-1.105) |  |
| **Hypertension** |  |  |
| Yes | 1.046 (1.006-1.088) | 1.000 |
| No | 1.044 (0.978-1.115) |  |
| Unknown | NA |  |
| Missing | NA |  |
| **Diabetes** |  |  |
| Yes | 1.105 (1.048-1.164) | 1.000 |
| No | 1.025 (0.979-1.073) |  |
| Borderline | 1.074 (0.812-1.422) |  |
| Unknown | NA |  |
| **CKD stage** |  |  |
| Stage 1 | NA | 1.000 |
| Stage 2 | NA |  |
| Stage 3a | 1.044 (0.980-1.113) |  |
| Stage 3b | 1.044 (0.961-1.135) |  |
| Stage 4 | NA |  |
| Stage 5 | 0.931 (0.802-1.081) |  |
| **Smoking history** |  |  |
| Current | 1.019 (0.931-1.115) | 1.000 |
| Former | NA |  |
| Never | 1.046 (0.999-1.096) |  |
| Not available | 1.328 (0.926-1.905) |  |
| **Drinking history** |  |  |
| Heavy drinker | 1.007 (0.943-1.075) | 1.000 |
| Low-to-moderate drinker | 1.086 (1.030-1.144) |  |
| Non-drinker | 1.052 (0.990-1.119) |  |
| Not available | 1.013 (0.952-1.078) |  |

Data were presented as hazard ratios (95% CIs) with adjustment of age (categorial), sex, race, education, marital status, smoking history, drinking history, dietary intakes during the past 24 hours (continuous), body mass index (categorial), hypertension, diabetes, albumin (categorial), albumin/globulin ratio (categorial), urinary albumin level (continuous), chronic kidney diseases stages (categorial) as well as National Health and Nutrition Examination Survey cycle.
