# Supplementary material for: Nonlinearity association between hyperuricemia and all-cause mortality in patients with chronic kidney disease
Source: Sci Rep. 2024 Jan 5;14:673. doi: 10.1038/s41598-023-51010-6 (PMC10770354; doi:10.1038/s41598-023-51010-6)
Supplement: Supplementary file 3 — Supplementary Information 3. [file 41598_2023_51010_MOESM3_ESM.docx]

**Supplementary Table 3: Hazards of mortality among 8861 CKD patients after excluding individuals who died within 2 years of follow-up.**

| **Mortality** | **Serum Uric Acid Levels** | | | | | ***P* for trend** | **Per serum uric acid**  **SD increment** |
| --- | --- | --- | --- | --- | --- | --- | --- |
|  | **≤4.428 mg/dL (Q1)** | **4.500 mg/dL -5.360 mg/dL (Q2)** | **5.400 mg/dL -6.293 mg/dL (Q3)** | **6.300 mg/dL -7.225 mg/dL (Q4)** | **≥7.300 mg/dL (Q5)** |  |  |
| Crude model | 1 (reference) | 1.465 (1.238-1.733) | 1.489 (1.258-1.763) | 1.689 (1.431-1.993) | 2.459 (2.098-2.882) | <0.001 | 1.335 (1.275-1.397) |
| Model 1 | 1 (reference) | 1.126 (0.975-1.301) | 1.012 (0.860-1.190) | 1.145 (0.969-1.352) | 1.503 (1.283-1.760) | <0.001 | 1.174 (1.111-1.241) |
| Model 2 | 1 (reference) | 1.048 (0.901-1.219) | 0.970 (0.818-1.152) | 1.008 (0.853-1.192) | 1.216 (1.007-1.468) | 0.031 | 1.083 (1.017-1.153) |

Crude model: without adjustment.

Model 1: adjusted for age (categorial) and sex.

Model 2: adjusted for model 1 plus race, education, marital status, smoking history, drinking history, dietary intakes during the past 24 hours (continuous), body mass index (categorial), hypertension, diabetes, albumin (categorial), albumin/globulin ratio (categorial), urinary albumin level (continuous), chronic kidney diseases stages (categorial) as well as National Health and Nutrition Examination Survey cycle.
