# Supplementary material for: Nonlinearity association between hyperuricemia and all-cause mortality in patients with chronic kidney disease
Source: Sci Rep. 2024 Jan 5;14:673. doi: 10.1038/s41598-023-51010-6 (PMC10770354; doi:10.1038/s41598-023-51010-6)
Supplement: Supplementary file 4 — Supplementary Information 4. [file 41598_2023_51010_MOESM4_ESM.docx]

Supplementary Table 4: **Hazards of mortality among 9251 CKD patients after excluding individuals with missing or non-available values.**

| **Mortality** | **Serum Uric Acid Levels** | | | | | ***P* for trend** | **Per serum uric acid**  **SD increment** |
| --- | --- | --- | --- | --- | --- | --- | --- |
|  | **≤4.428 mg/dL (Q1)** | **4.500 mg/dL -5.360 mg/dL (Q2)** | **5.400 mg/dL -6.293 mg/dL (Q3)** | **6.300 mg/dL -7.225 mg/dL (Q4)** | **≥7.300 mg/dL (Q5)** |  |  |
| Crude model | 1 (reference) | 1.497 (1.290-1.738) | 1.525 (1.285-1.809) | 1.747 (1.490-2.049) | 2.510 (2.144-2.938) | <0.001 | 1.342 (1.284-1.402) |
| Model 1 | 1 (reference) | 1.158 (1.018-1.316) | 1.041 (0.877-1.235) | 1.176 (1.005-1.376) | 1.521 (1.300-1.779) | <0.001 | 1.177 (1.117-1.241) |
| Model 2 | 1 (reference) | 1.061 (0.921-1.221) | 1.021 (0.859-1.215) | 1.078 (0.923-1.259) | 1.251 (1.034-1.513) | 0.012 | 1.092 (1.023-1.166) |

Crude model: without adjustment.

Model 1: adjusted for age (categorial) and sex.

Model 2: adjusted for model 1 plus race, education, marital status, smoking history, drinking history, dietary intakes during the past 24 hours (continuous), body mass index (categorial), hypertension, diabetes, albumin (categorial), albumin/globulin ratio (categorial), urinary albumin level (continuous), chronic kidney diseases stages (categorial) as well as National Health and Nutrition Examination Survey cycle.
