# Supplementary material for: Nonlinearity association between hyperuricemia and all-cause mortality in patients with chronic kidney disease
Source: Sci Rep. 2024 Jan 5;14:673. doi: 10.1038/s41598-023-51010-6 (PMC10770354; doi:10.1038/s41598-023-51010-6)
Supplement: Supplementary file 5 — Supplementary Information 5. [file 41598_2023_51010_MOESM5_ESM.docx]

**Supplementary Table 5: Associations of serum uric acid level with mortality when considering the influence of drug usage.**

| **Mortality** | ***P* for trend** | **Per serum uric acid**  **SD increment** |
| --- | --- | --- |
|  |  |  |
| **Crude model** | <0.001 | 1.280 (1.235-1.326) |
| **Model 1** | <0.001 | 1.151 (1.104-1.200) |
| **Model 2** | 0.014 | 1.073 (1.022-1.127) |
| **Model 3** | 0.021 | 1.071 (1.020-1.124) |

Crude model: without adjustment.

Model 1: adjusted for age (categorial) and sex.

Model 2: adjusted for model 1 plus race, education, marital status, smoking history, drinking history, dietary intakes during the past 24 hours (continuous), body mass index (categorial), hypertension, diabetes, albumin (categorial), albumin/globulin ratio (categorial), urinary albumin level (continuous), chronic kidney diseases stages (categorial) as well as National Health and Nutrition Examination Survey cycle.

Model 3: adjusted for model 2 plus drug usage.
