# Supplementary material for: Nonlinearity association between hyperuricemia and all-cause mortality in patients with chronic kidney disease
Source: Sci Rep. 2024 Jan 5;14:673. doi: 10.1038/s41598-023-51010-6 (PMC10770354; doi:10.1038/s41598-023-51010-6)
Supplement: Supplementary file 6 — Supplementary Information 6. [file 41598_2023_51010_MOESM6_ESM.docx]

**Supplementary Table 6: Associations of serum uric acid level with mortality among 9891 CKD patients after considering the influence of cardiovascular diseases and HbA1c level.**

| **Mortality** | **Serum Uric Acid Levels** | | | | | ***P* for trend** | **Per serum uric acid**  **SD increment** |
| --- | --- | --- | --- | --- | --- | --- | --- |
|  | **≤ 4.521 mg/dL (Q1)** | **4.600 mg/dL - 5.400 mg/dL (Q2)** | **5.453 mg/dL - 6.293 mg/dL (Q3)** | **6.300 mg/dL - 7.300 mg/dL (Q4)** | **≥ 7.318 mg/dL (Q5)** |  |  |
| **Crude model** | 1 (reference) | 1.400 (1.192-1.646) | 1.413 (1.204-1.659) | 1.742 (1.469-2.067) | 2.504 (2.149-2.917) | <0.001 | 1.291 (1.243-1.341) |
| **Model 1** | 1 (reference) | 1.117 (0.963-1.295) | 1.016 (0.864-1.195) | 1.176 (0.994-1.391) | 1.590 (1.357-1.863) | <0.001 | 1.160 (1.109-1.214) |
| **Model 2** | 1 (reference) | 1.051 (0.904-1.222) | 0.979 (0.835-1.148) | 1.036 (0.874-1.228) | 1.301 (1.078-1.569) | 0.014 | 1.087 (1.034-1.144) |
| **Model 3** | 1 (reference) | 1.038 (0.893-1.207) | 0.972 (0.824-1.147) | 1.026 (0.866-1.215) | 1.269 (1.056-1.525) | 0.032 | 1.077 (1.022-1.134) |

Crude model: without adjustment.

Model 1: adjusted for age (categorial) and sex.

Model 2: adjusted for model 1 plus race, education, marital status, smoking history, drinking history, dietary intakes during the past 24 hours (continuous), body mass index (categorial), hypertension, diabetes, albumin (categorial), albumin/globulin ratio (categorial), urinary albumin level (continuous), chronic kidney diseases stages (categorial) as well as National Health and Nutrition Examination Survey cycle.

Model 3: adjusted for model 2 plus cardiovascular diseases and HbA1c (continuous).
